# Acetate Production from Cafeteria Wastes and Corn Stover Using a Thermophilic Anaerobic Consortium: A Prelude Study for the Use of Acetate for the Production of Value-Added Products

**DOI:** 10.3390/microorganisms8030353

**Published:** 2020-03-02

**Authors:** Aditi David, Abhilash Kumar Tripathi, Rajesh Kumar Sani

**Affiliations:** 1 Department of Chemical and Biological Engineering, South Dakota School of Mines and Technology, Rapid City, SD 57701, USA; aditi.david@mines.sdsmt.edu (A.D.); abhilashkumar.tripathi@mines.sdsmt.edu (A.K.T.); 2 BuG ReMeDEE Consortium, South Dakota School of Mines and Technology, Rapid City, SD 57701, USA

**Keywords:** acetogenic, thermophilic consortium, solid organic wastes, food and paper waste, *Yarrowia lipolytica*

## Abstract

Efficient and sustainable biochemical production using low-cost waste assumes considerable industrial and ecological importance. Solid organic wastes (SOWs) are inexpensive, abundantly available resources and their bioconversion to volatile fatty acids, especially acetate, aids in relieving the requirements of pure sugars for microbial biochemical productions in industries. Acetate production from SOW that utilizes the organic carbon of these wastes is used as an efficient solid waste reduction strategy if the environmental factors are optimized. This study screens and optimizes influential factors (physical and chemical) for acetate production by a thermophilic acetogenic consortium using two SOWs—cafeteria wastes and corn stover. The screening experiment revealed significant effects of temperature, bromoethane sulfonate, and shaking on acetate production. Temperature, medium pH, and C:N ratio were further optimized using statistical optimization with response surface methodology. The maximum acetate concentration of 8061 mg L^−1^ (>200% improvement) was achieved at temperature, pH, and C:N ratio of 60 °C, 6, 25, respectively, and acetate accounted for more than 85% of metabolites. This study also demonstrated the feasibility of using acetate-rich fermentate (obtained from SOWs) as a substrate for the growth of industrially relevant yeast *Yarrowia lipolytica*, which can convert acetate into higher-value biochemicals.

## 1. Introduction

The need for sustainable energy generation in recent years has increased interest in microbial processes for the production of fuels and value-added products. Concurrently, the search for finding alternative low-cost substrates has also increased and the use of substrates that create direct competition with the food resources has been discouraged [1]. Commonly used substrates for microbial fermentation are simple sugars such as glucose and sucrose. Solid organic wastes (SOWs) such as food and paper waste, and agricultural wastes (such as corn stover) produced by anthropogenic activities, are abundant substrates that can be converted to intermediate or end-use bioproducts using microbial metabolism [2,3]. An enriched microbial consortium consisting of different microbial groups with diverse functions can degrade these SOWs into value-added end products or platform chemicals. Within the microbial consortium, hydrolytic enzymes produced by microbes convert the complex substrates into simpler sugars and organic acids. These intermediate organic acids often called as volatile fatty acids (VFAs) can be further utilized for microbial production of higher-value chemicals and thus can replace sugar substrates in industrial fermentations. VFAs are attractive substrates because they can be produced from a variety of organic wastes fermentation [4]. Therefore, the use of VFAs as carbon sources seems to be a feasible strategy for cost-effective microbial productions. Acetic acid or acetate is the most desirable VFA as it can serve as an alternate and sole source of carbon for microorganisms and can be used at comparatively higher concentrations to replace sugar substrates. The global demand of acetic acid is 14.3 billion pounds per year and it can be employed in the production of photographic film (cellulose acetate), wood glue (polyvinyl acetate), and synthetic fibers (triacetate cellulose) used for textile, carpets, and cigarette filters, among other products [5,6]. In addition to anaerobic fermentation by the acetogenic microbial consortium, acetic acid is also produced using chemical processes (e.g., methanol carbonylation, ethylene oxidation, and alkane oxidation) [7]. In the United States, the price of acetate ($350−$450 per ton) is lower than the price of glucose ($500 per ton) [8]; however, it is possible to obtain high titers of acetate inexpensively using renewable, and abundant feedstocks such as SOWs. 

The microbial consortium carrying out thermophilic anaerobic digestion (TAD) comprise a significant fraction of acetogenic (acetate-producing) bacteria such as *Acetomicrobium, Clostridium*, and *Acetobacter sp.* which can convert SOW to acetate more rapidly and without any chemical or physical pretreatment [9]. The use of a TAD system is advantageous because the high-temperature fermentation process increases acetate end product formation and prevents the formation of other VFAs such as butyrate [10]. Thermophilic bacteria are also reported to simultaneously utilize mixed hexose and pentose sugars which could be beneficial when the SOW used is a lignocellulosic substrate [10,11]. Thermophilic conditions have additional benefits of high substrate degradation rate, pathogen removal, and efficient heat utilization of SOWs. The thermophilic microbial consortium also harbors distinct microbial species that possess metabolic functions related to biomass degradation and utilization [12,13]. Therefore, a TAD system fed with SOWs can be rewired to an acetate-producing system by inhibition of methanogenesis. This results in simultaneous enrichment of a thermophilic acetogenic consortium (TAC) which primarily produces acetate. 

Most prior studies have optimized the factors leading to high total VFA productions [4,14,15] for fuels and chemical applications. All these research endeavors are important considering that raw material alone constitutes 40%–80% of biofuel production costs. Biofuels made from VFAs derived from waste organic biomass potentially offer significant economic advantages [16]. To reduce production costs, some pure culture studies have been conducted and reviewed using SOWs [17,18,19]. However, the literature is limited regarding thermophilic anaerobic fermentation processes using mixed consortia that would use SOWs and produce a higher fraction of acetate (in the VFA pool). Though the control of metabolite is difficult in mixed culture fermentations compared to pure culture counterparts, however, mixed culture fermentations are advantageous due to the absence of sterilization requirements, a stable operation when designed on proper ecological selection principles, the potential for stable continuous operation, and adaptive capacity to variations in feed or conditions [20]. If optimized, mixed culture fermentation processes have a great chance to outcompete pure culture fermentations [17]. In addition, the production of a higher fraction of a single VFA (i.e., acetate in this study) is desirable for simplifying downstream recovery processes. Among the different VFAs, acetic acid has the highest market size, with 3,500,000 tons/y and a price per ton of $800 [21]. The production of predominant acetate together with methane in extreme-thermophilic (70 °C) mixed culture fermentation was first studied by Zhang et al. [12]. However, they used pure glucose as a substrate to achieve >90% acetate in the fermentate’s metabolite pool. In contrast, our study uses completely SOWs to produce acetate-rich fermentate. Knowledge of environmental factors affecting the acetate production from thermophilic acetogenic consortium is of considerable importance for the efficient conversion of SOWs into acetic acid at an industrial scale. Therefore, this work aims at developing a microbial consortium and maximizing acetate production using two SOWs—cafeteria (food and paper) wastes and corn stover—as a substrate. Initial screening experiments were conducted using two-level factorial design to screen for most influential parameters affecting the conversion of cafeteria and corn stover waste into acetic acid. Further, optimization of the process conditions, i.e., incubation temperature, pH, and C:N ratio, was done to understand their impact on acetic acid production at individual and interactive levels using statistical optimization with response surface methodology (RSM). Statistical optimization is a very effective tool in determining the process parameter values for increasing the desired product yield because it takes into account the interaction effects of the process parameters tested [22,23]. 

Various industrially relevant microorganisms can naturally utilize acetic acid and convert it into valuable bioproducts [24,25,26,27,28]. Advancement in the field of microbial genetics and metabolic engineering has furthered the range of bioproducts synthesized and substrates that can be utilized by microorganisms [29,30,31]. Research efforts have also been directed toward increasing the substrate uptake and its utilization. This is important to achieve higher bioproduct yields and productivity and, therefore, can improve the overall economics of industrial microbial processes. An industrially relevant host organism is the oleaginous yeast—*Yarrowia lipolytica*—which has gained a lot of attention recently for the production of lipids using various pure substrate (e.g., glucose, fructose, lactose, sucrose, glycerol, and xylose) and raw feedstocks (e.g., olive oil mill waste, whey, waste cooking oil, and animal fats) [26,32]. Oleaginous yeasts have a distinctive ability to convert certain organic acids directly to acetyl-CoA—the central intermediate of lipid biosynthesis—by acetyl coenzyme A synthetase. Acetyl-CoA is subsequently utilized in fatty acid (FA) synthesis and results in lipid accumulation [33]. Recent studies have also explored the potential of native and engineered *Y.lipolytica* to synthesize products other than lipids including itaconic acid [34], erythritol [35], citric acid [36], carotenoids [37], and polyhydroxyalkanoates [38]. So far, most studies on bioproduct synthesis by oleaginous microorganisms like *Yarrowia lipolytica* have been carried out with glucose as a carbon source. Published literature on volatile fatty acids (derived from different SOWs) as a sole carbon source for this yeast is scarce and no previous study has attempted to convert these low-cost volatile fatty acids into itaconic acid (a dicarboxylic acid), which is industrial chemical serving as a precursor of polymers used in plastics, adhesives, and coatings. It is also one of the “top 12” building block chemicals listed by the United States Department of Energy [39]. We are currently engineering a *Yarrowia lipolytica* strain to increase its ability to uptake acetate as well as upregulate the metabolic pathways that increase acetate assimilation and divert its metabolic flux toward itaconic acid production (unpublished work). For the current study, we studied the growth of the wild-type *Y.lipolytica* strain as a proof-of-concept for growing the engineered strain on the acetate-rich-fermentate in our future work. So, in addition to optimizing acetate production through anaerobic co-fermentation of cafeteria waste and corn stover, this study analyzes the feasibility of using this acetate-rich fermentate for growth of *Y.lipolytica*.

## 2. Materials and Methods 

### 2.1. Substrate 

Mixed SOWs (corn stover and cafeteria wastes) were used as substrates for all optimization experiments. Corn stover was kindly provided by Dr. K. Muthukumarappan from South Dakota State University, Brookings, SD. It was crushed using a blender and sieved through sieve between 15- and 10- mm pores before being used as a substrate. Cafeteria wastes consisting of paper and food wastes were obtained from the South Dakota of Mines and Technology (SDSMT) cafeteria. The composition of cafeteria waste varies depending on many factors such as region and season. So to reduce some of this bias, cafeteria wastes were collected on 3 separate days over a span of 3 months, homogenized, and reduced in size using a mechanical mixer and stored at −20 °C until use. 

### 2.2. Growth Medium and Experimental Set-Up

A modified basal anaerobic medium was used for inoculum development and comprised of (g/L): K_2_HPO_4_, 0.3; KH_2_PO_4_, 0.3; NaCl, 0.1; CaCl_2_, 0.05; NH_4_Cl, 1.0; MgCl_2_·6H_2_O 0.5; KCl, 0.3; cysteine HCl, 0.5; yeast extract, 0.05 and Na_2_S·9H_2_O, 0.003. NaHCO_3_ and Nitsch trace element solution were added to the medium to a final concentration of 20 mM and 2.5 mL per liter, respectively. All the experiments were performed in duplicates in 500 mL serum bottles with 200 mL working volume consisting of 10% *v/v* inoculum in anaerobic basal medium. The serum bottle reactors were autoclaved at 121 °C and 15 psi for 20 min prior to inoculation. A 50 mM solution of 2-bromoethanesulfonate (BES) was added to inhibit methanogenesis and enhance acetate production in different experimental runs according to the design of experiments. Following the addition of BES and inoculum, the serum bottle reactors were sealed with butyl rubber stoppers, crimped with aluminum crimps, and purged with nitrogen gas for 20 min to establish anaerobic conditions. They were incubated at different temperatures and in the presence or absence of shaking at 100 rpm according to the design of experiments. 

### 2.3. Inoculum Development 

The inoculum used was a combination of two different types of anaerobic sludge collected from the Wastewater Reclamation Plant (Rapid City, SD, USA) and from a lab-scale anaerobic digester fed with food, paper, and lignocellulosic wastes (at the Chemical and Biochemical Engineering Department, SDSMT, Rapid City, SD, USA). The inoculum was prepared by mixing the two anaerobic sludges in equal amounts (50/50 *v/v*) and adjusting the pH to 6 with 1 M HCl while flushing with N_2_ to ensure anaerobic conditions. The inoculum was sieved through a 2 mm net after collection to remove large particles. The acetogenic consortia were developed and maintained in the lab using sub-culturing techniques as described by David and coll. (2018) [40]. Briefly, one-gram volatile solids (VS) of mixed SOW (containing equal amounts of corn stover and cafeteria wastes) was added to the modified anaerobic basal medium and the bottles were incubated at 60 °C and 100 rpm. When acetate production reached a stable level, 10% (*v/v*) of the actively growing anaerobic culture was transferred into fresh basal media (200 mL) containing another 1 g of mixed waste. After 10 serial transfers, a thermophilic acetogenic consortium was obtained that produced acetate using corn stover and cafeteria wastes as carbon and energy source. All experiments and chemical analyses were done in duplicates and the value presented is an average of the two values from the duplicate set up. The microbial community analysis of the thermophilic consortium while shifting from biogas production to primarily acetate production is being studied using Illumina sequencing and will be part of a separate manuscript (in preparation).

### 2.4. Analytical Methods

Total solids (TS) and volatile solids (VS) of the substrate and inoculum were measured according to APHA standard methods [41]. The cellulose, hemicellulose, and lignin content were measured according to NREL analytical methods [42]. Elemental analysis was done by Atlantic Microlab, Norcross, GA. Liquid samples from the serum bottle reactors were analyzed for three major volatile fatty acids (VFAs) (acetate, propionate, and butyrate) at regular time intervals over a course of 8 days or until the acetate production ceased increasing. The liquid samples were centrifuged at 10,000 rpm for 10 min to remove suspended solids and biomass prior to VFA measurement. VFA concentrations were determined using HPLC equipped with Aminex HPX-97 H column with 0.005 N sulfuric acid as the mobile phase. Acetate percent was calculated by dividing the concentration of acetate (mg/L) in the reactor liquid by the total concentration of the measured VFAs (i.e., the sum of acetate, propionate, and butyrate concentrations in mg/L). Carbon to nitrogen ratio was calculated using the following equation [40,43]:

(1) $$C:\;N\;=\;\frac{{\left(\mathrm{VS}*\mathrm{TOC}\right)}_{CFW}\;+\;{\left(\mathrm{VS}*\mathrm{TOC}\right)}_{CS}}{{\left(\mathrm{VS}*\mathrm{TN}\right)}_{CFW}\;+\;{\left(\mathrm{VS}*\mathrm{TN}\right)}_{CS}}$$

where TOC = total organic carbon (%VS) and TN = total nitrogen (%VS)

### 2.5. Design of Experiment for Optimization of Acetate Production 

#### 2.5.1. Selection of Influential Factors 

Ten factors that could influence acetate fermentation and growth of the acetogenic consortium were selected and tested using two-level factorial design in Design Expert Version 12.0.6.0 (Stat-Ease Inc., Minneapolis, Minnesota, MN, USA) statistical software. In a two-level factorial experiment each independent variable, i.e., a “factor” is investigated at two discrete possible values or “levels”. This is beneficial for estimating the main effects as well as the interaction effects of the factors tested. The 10 factors chosen are shown in Table 1. Thirty-two different experimental runs were performed. The factors included physical parameters (temperature, shaking), chemical factors (pH and C:N ratio), and inorganic medium components (NaHCO_3_, NH_4_Cl, MgCl_2_.6H_2_O, and KCl). The impact of BES (methanogen inhibitor) and Na_2_S.9H_2_O (to give basal media reducing power) addition was also investigated. With a signal-to-noise ratio of 3, the power obtained for the statistical design was 99.9%. This was greater than the minimum recommended (>80%) for the effects that were to be detected. A low substrate load was used to dilute the inhibitory molecules that may be present in either cafeteria waste or corn stover, and that could potentially impact acetate fermentation by the newly developed consortium. Therefore, two-gram VS was used as a substrate for all the runs of this screening experiment. The factors that positively affected acetate concentration in the test runs were either selected for further optimization through RSM or were incorporated in all the RSM runs.

#### 2.5.2. Response Surface Methodology for Statistical Optimization of Influential Factors for Acetate Production

RSM is a widely used statistical tool for modeling or analyzing a process in which the parameters which affect the process are called independent variables, whereas the responses are called dependent variables [44]. The objective for the RSM method is to optimize the response of interest which is affected by various variables, i.e., factors incorporated in the RSM design [45]. Response surface methodology (RSM) was used to optimize factors having a significant effect on acetate production by anaerobic fermentation of mixed SOW. Three factors (independent variables)—temperature, pH, and C:N ratio—were chosen for statistical optimization of acetate production (dependent variable) in this study. A substrate load of 5 g VS was used for all the experimental runs. A 2^3^ factorial central composite design with 14 non-center points and six replications at the center points (α = 1.68) yielded a total of 20 experimental runs. Each factor/variable was set to 5 levels: +α and −α (axial points), +1 and −1 (factorial points), and the center point (Table 2). The Design Expert (Version 12.0.6.0) statistical software was used to analyze the results of the experimental design. Response was recorded in terms of acetate concentration in mg L^−1^. Response data generated by the experimental runs are recorded in Table 3. It was subjected to analysis of regression through RSM to solve multivariate equations. The behavior of the system was explained by the following quadratic equation:

(2) $$Y\;=\;\beta_{0}+\sum \beta_{i}X_{i}+\sum \beta_{ii}X_{i}^{2}+\;\sum \beta_{ij}X_{i}X_{j}$$

where Y is the predicted response (acetate concentration, mgL^-1^), X represents individual factors being considered in the design (in this case temperature, pH, and C:N ratio), β_0_ is the intercept, β_i_ linear effect coefficient, β_ii_ is the quadratic effect coefficient, and β_ij_ is interaction effect coefficient. The significance of each effect was estimated by analysis of variance with *p*-values < 0.05, i.e., above a 95% confidence interval. The Statistical Design Expert software was applied for multiple regression analysis and to create the plots of the obtained data. Each experiment was performed in triplicates and the data presented is mean of the triplicate values.

### 2.6. Growth of Yarrowia Lipolytica on SOW-Derived Acetate-Rich Anaerobic Fermentation Product

The yeast strain *Y.lipolytica* strain Po1f was used to demonstrate the feasibility of using acetate-rich fermentate derived from optimized anaerobic fermentation of SOWs. *Y.lipolytica* (American Type Culture Collection (ATCC) no. MYA 2613) was procured from American Type Culture Collection (ATCC), USA. It was maintained as a glycerol stock at −80 °C prior to growth study. The anaerobically fermented product of SOW was centrifuged at 10,000 rpm for 10 min to precipitate biomass and suspended/undigested solids. The acetate and other VFA concentrations were measured in the supernatant obtained after filtration through a 0.2 µM membrane. This acetate-rich VFA supernatant was used as a substrate for *Y.lipolytica*’s growth without additional nutrients. The final acetate concentrations of 2, 4, 6, and 8 g L^−1^ were adjusted by diluting with sterile de-ionized (DI) water. The pH was adjusted to 6.0 and the growth experiments were conducted in 500 mL Erlenmeyer flasks with a 200 mL working volume of fermentate and inoculum.

*Y.lipolytica* was precultured from glycerol stocks by inoculating 200 mL of YPD broth (20 g L^−1^ peptone, 10 g L^−1^ yeast extract, 20 g L^−1^ glucose) in Erlenmeyer flasks and incubated at 28 °C (200 rpm) until the late exponential growth phase (24 h–26 h). For growth study, this preculture was used to inoculate 200 mL VFA supernatant to a starting OD_600_ of 0.2. A control experiment with glucose (2 g L^−1^) as the sole carbon source was also set up. Growth of *Y.lipolytica* was assessed by measuring biomass density in terms of dry cell weight (DCW). DCW was determined by vacuum filtering 5 mL of culture in the stationary growth phase through a pre-dried and pre-weighed nitrocellulose filter, and the sample was washed several times. The samples were dried to a constant weight before analyzing DCW. All growth experiments were performed in triplicates. In the presence of high VFA concentration (>6 g L^−1^), the batch cultures were incubated for 20 days in order to accommodate for adaptation of the yeast (lag phase of growth) to unfavorable culture conditions. All other batch cultures having different concentrations of acetate were cultivated until a measurable biomass production was observed.

## 3. Results and Discussion

### 3.1. Substrate and Inoculum Characteristics

The substrate and inoculum characteristics are shown in Table 3. The TS and VS concentrations of the cafeteria waste was 28.3% ± 0.6% and 23.2% ± 0.7%, respectively, whereas corn stover contained 94.8% ± 0.6% TS and 89.5% ± 0.8% VS. The cellulose, hemicellulose, and lignin content of the corn stover used in this study was 39.4%, 32.1%, and 12.8%, respectively. The inoculum had the TS and VS content of 4.3% ± 0.5% and 2.6% ± 1%, respectively. Sub-culturing the inoculum with the same wastes as used for the experimental batch runs allowed for acclimatization of the thermophilic microbial consortium which is favorable for achieving higher acetate production [46,47,48,49]. Though the cafeteria waste had lower VS/TS percentage, it consisted of a higher amount of easily biodegradable organic compounds which made it a more easily hydrolyzable substrate for acetate production compared to corn stover. The C:N ratio of cafeteria waste, however, was substantially lower than corn stover. Therefore, apart from being an abundant, easily accessible, and inexpensive substrate, corn stover with a considerably higher C:N ratio was a suitable co-substrate for cafeteria waste. Using two substrates with a significant difference in their C:N ratio allowed adjustment of the C:N ratio to different values needed for the design of this study.

### 3.2. Optimization of Acetate Production

The microbial consortium during anaerobic fermentation can produce various gaseous (methane, hydrogen, carbon dioxide) and liquid products (acetate, propionate, butyrate, ethanol, lactate) in addition to increasing their biomass. Therefore, to increase the production of acetate, several factors were studied to analyze their impact and find the optimum values for the most influential factors for enhanced acetate production.

#### 3.2.1. Selection of Influential Factors

**Figure 1:**
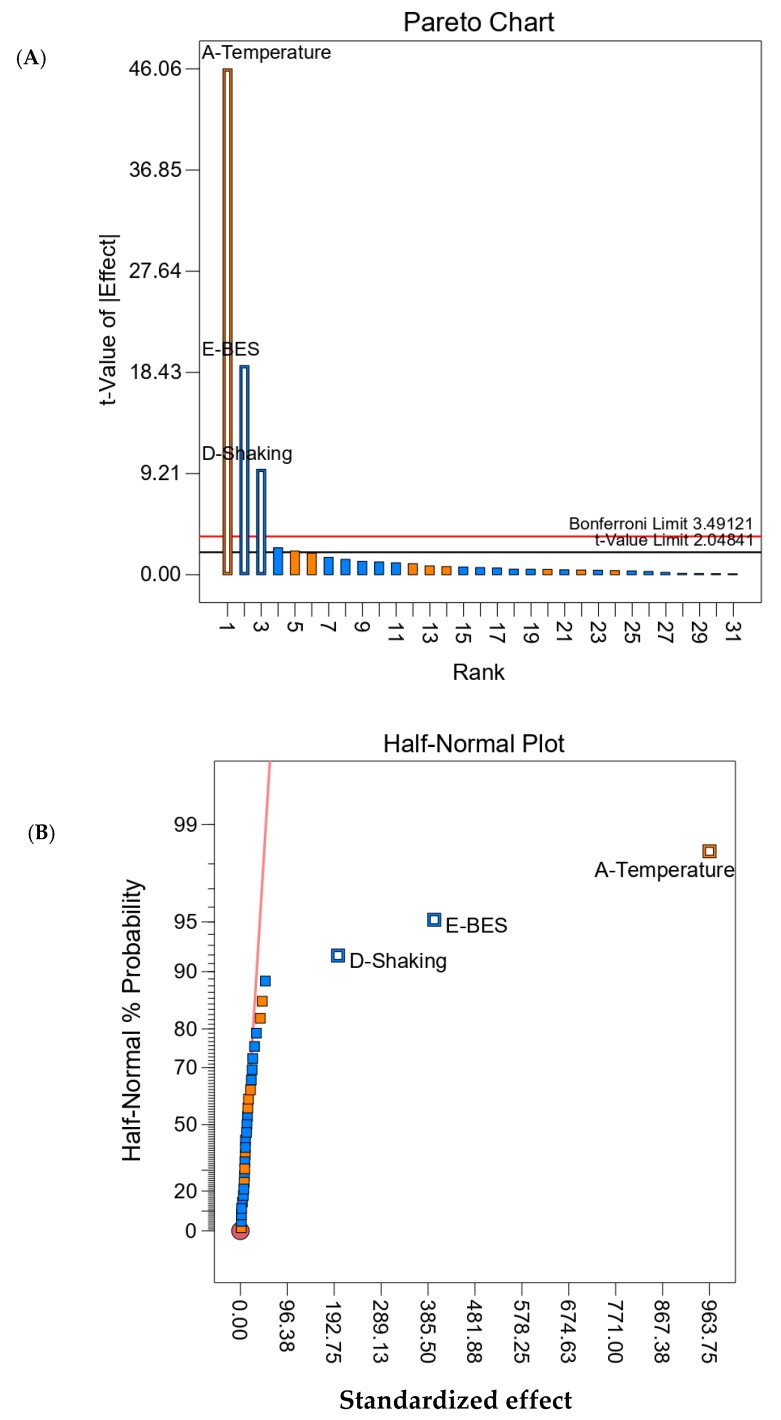
(**A**) Pareto charts and (**B**) half-normal plot for two-level factorial design. Above the Bonferroni limit (3.49121) the effects are significant. Effects in between the t-limit (2.04841) and Bonferroni limit may possibly be significant. Effects below the t-value limit are insignificant.

Based on previous work and the available literature [21,46,50], 10 factors that could influence acetate fermentation and growth of the acetogenic consortium were selected. The influence of these 10 factors, i.e., temperature, pH, C:N ratio, shaking (100 rpm), BES (50mM), NaHCO_3_, NH_4_Cl, MgCl_2_.6H_2_O, KCl, and Na_2_S.9H_2_O, was investigated using two-level factorial design. Two-level factorials can be used for screening many factors to find the significant few [21,46]. Parameters, experimental runs, and response (acetate concentration) of the experimental design are shown in Supplementary Table S1. The acetate concentration ranged between 483 mg L^−1^ and 2148 mg L^−1^ for the different experimental runs. In order to check the influence of the aforementioned factors, half-normal plots and pareto charts were analyzed (Figure 1). Pareto charts indicated that out of 10 factors tested, three factors, i.e., temperature, BES, and shaking, had the most significant effect on acetate production. The analysis of variance (ANOVA) for the selected factorial model with these three significant factors is given in Supplementary Table S2 and the Fit statististics are given in Supplementary Table S3.

Average acetate production at an incubation temperature of 60 °C (1739.44 mg L^−1^) was more than two-folds higher than the acetate production at 45 °C (775.68 mg L^−1^). Previous studies have also shown the prominent effect of temperature on acetate production [4,12,51]. For both the incubation temperatures, the presence of BES and shaking at 100 rpm had a positive influence on acetate production. On average, the acetate production increased 1.4 and 1.2 times in the presence of BES and shaking, respectively. The effect of shaking was more pronounced at 45 °C (1.3 times improvement in average acetate concentration), while the effect of BES addition was more significant at 60 °C (1.6 times improvement in average acetate concentration). BES is a potent inhibitor of acetoclastic methanogenesis in thermophilic anaerobic systems [52,53]. It has previously shown to completely the inhibit activity of methanogens at a concentration of 50 mM (also used in this study) [52,54]. Nevertheless, the literature shows contrasting effects of BES on inhibition of methanogens and VFA production which appear to be correlated to organic substrate loading [21,46,54]. In a recent study conducted by Lukitawesa and coll. (2020), BES addition showed more pronounced effects at lower substrate loading than higher substrate loading [46]. It was not surprising that the third most influential factor according to this study was shaking because previous studies have also reported enhanced acetate production under shaking conditions than static conditions [51,55,56].

As the two temperatures chosen for the initial screening spanned a wide range (45 °C and 60 °C), temperature was selected as one of the factors for further statistical optimization though the RSM design. pH and C:N ratio were the other two factors chosen for better tuning of the optimum values as both have been reported to a exert strong effect on acetate production in previous studies [21,46,57,58]. The effect of pH and C:N ratio in the two-level factorial design in this study could have been masked by the stronger individual and combined effect of temperature, BES addition and shaking. To eliminate this masking effect and to better understand the effect of pH and C:N ratio, BES and shaking conditions were kept the same in all the runs of the RSM design.

#### 3.2.2. Optimization of Influential Factors for Acetate Production Using RSM

Based on the results of the above-mentioned screening experiment, the effects of temperature, different controlled pH values, and initial C:N ratio were investigated in the second experiment (i.e., optimization using RSM). The response parameter (acetate in mg L^−1^) was the same as in the first experiment. A quadratic model based on the central composite design was developed in RSM to find optimum parameter values and study the combined effect of the variables—temperature, pH, and C:N ratio. The central composite design resulted in 20 runs and the actual (experimentally observed) as well as predicted responses with the residuals are presented in Table 4. The predicted and observed responses were analyzed by ANOVA. The main factor effects and two-factor interaction effects influencing the acetate production are shown in Table 5. A second-order regression equation provided the acetate concentration as a function of temperature, pH, and C:N ratio which is presented as a model equation for predicting acetate concentration. The model equation in terms of coded factors is as follows:

(3) $$Y\;=\;0.2661.5+256.82A-49.76B-8.64C-\;26.03\mathrm{AB}+7.47\mathrm{AC}+6.71\mathrm{BC}-545.25{\;A}^{2}-\;678.04B^{2\;}-246.43C^{2}$$

where Y, A, B, and C are acetate (mg L^−1^), temperature, pH, and C:N ratio, respectively.

**Figure 2:**
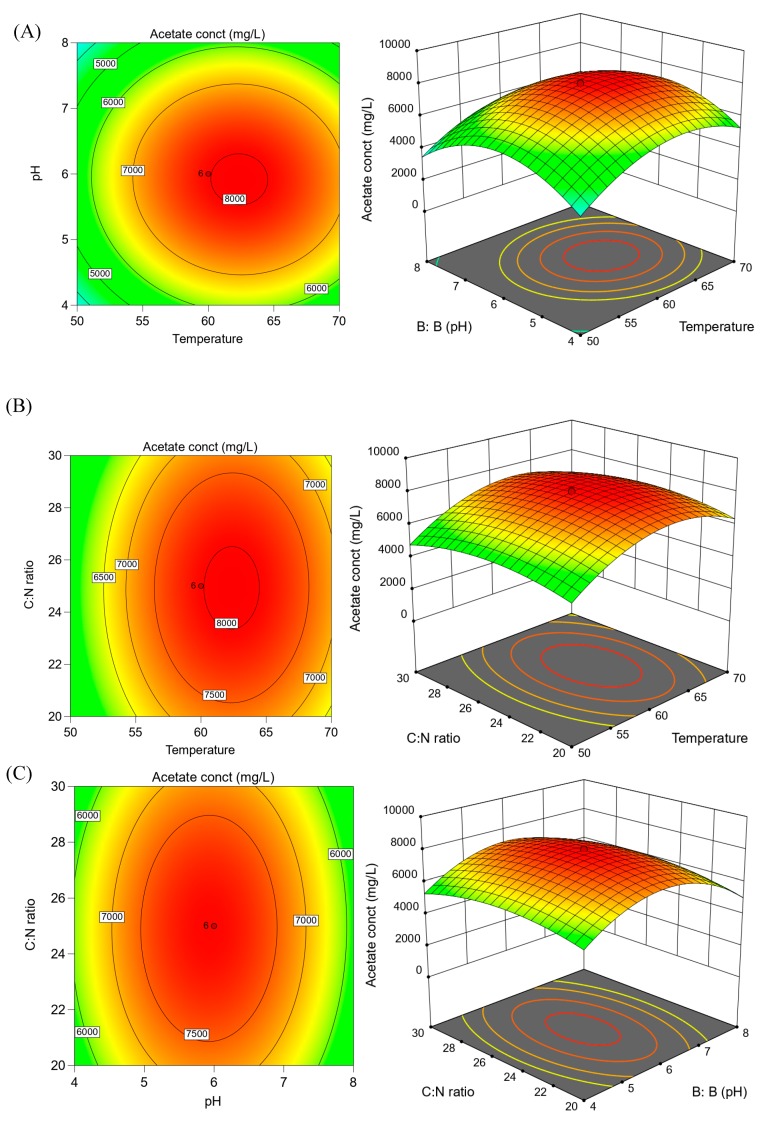
Three-dimensional response surface plots showing acetate concentration by thermophilic acetogenic consortium and interaction between: (**A**) pH and temperature; (**B**) C:N ratio and temperature; (**C**) C:N ratio and pH. The minimum and maximum values are represented by green and red color, respectively. The other colors represent values in between the minimum and maximum values. These plots and graphs were created for the pair-wise combination of the two factors while keeping the third factor at its optimum value.

The coded equation is useful for identifying the relative impact of the factors by comparing the factor coefficients. The equation in terms of coded factors can be used to make predictions about the response (acetate in mg L^−1^) for given levels of each factor. This equation was used to obtain contour plots and 3D response surface graphs which was used to predict optimum values of the three factors tested (Figure 2). These plots and graphs were created for the pair-wise combination of the two factors while keeping the third factor at its optimum value. 

**Figure 3:**
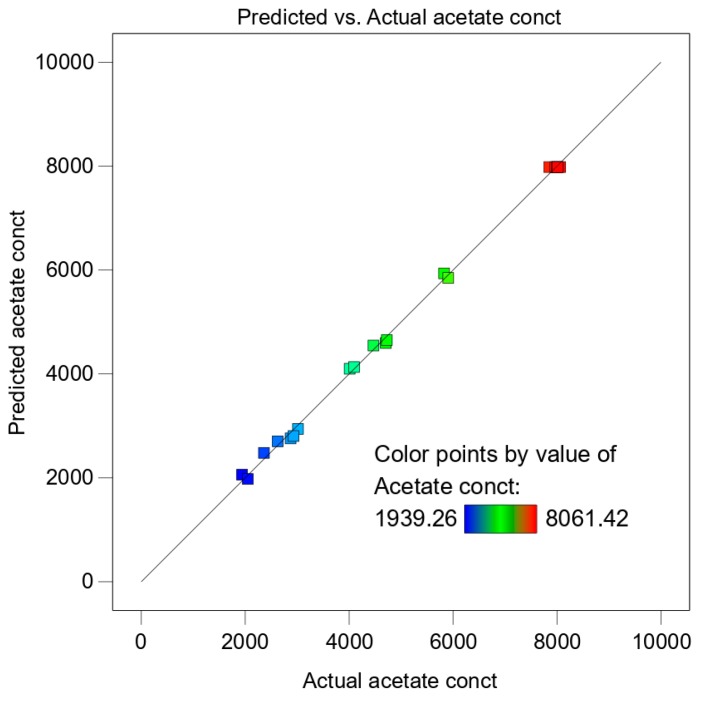
Surface response plot for predicted vs. actual values (acetate conct. in mg L^−1^). The squares represent the experimental values obtained from the runs. The concentration of the data values near the straight line shows a high correlation and adequate precision. The minimum value is denoted by a blue color, whereas the maximum value is denoted by a red color. The rest of the colors represent a range between the minimum and maximum value.

The analysis of variance (ANOVA) showed that the quadratic model was significantly based on *p*-values and an F-test (Table 5). The Model F-value of 753.01 implies the model is significant. There is only a 0.01% chance that an F-value this large could occur due to noise. Lack of fit F-value of 4.78 implies there is a 5.56% chance that a lack of fit F-value this large could occur due to noise. Lack of fit was not significant relative to the pure error which is good because we want the model to fit. The Model *p*-value of <0.0001 also indicated high model significance. P-values less than 0.05 indicate model terms are significant. In this case, A, B, A², B², and C² are significant model terms (Table 5). The Predicted R² of 0.9985 was in reasonable agreement with the Adjusted R² of 0.9972 (Supplementary Table S4). A low coefficient of variance value (C.V. %) of 2.45 indicated adequate precision and applicability of the model to navigate the design space. Adequate precision (signal-to-noise ratio) of greater than four was desirable and the model had a ratio of 69.743. All these evaluations confirmed that the model can be used for the prediction of maximum acetate production using our thermophilic acetogenic consortium. In the surface response plot (Figure 3), the concentration of the data points near the straight line also indicates high correlation and precision.

Effects of pH and temperature are shown in Figure 2A. pH and temperature are key parameters during acidogenic fermentation as it impacts both microbial growth and metabolism [4]. At the lowest pH and reaction temperature, acetate production was low. As the pH and temperature increased, the VFA production also increased gradually to reach a maximum value; it then decreased. The optimal temperature and pH for VFA production in this study were 60 °C and 6.0, respectively. This finding is consistent with our previous study where a temperature of 60 °C was found to be optimum for the thermophilic consortium carrying out anaerobic fermentation of food, paper, and lignocellulosic wastes [40]. The positive effect of slightly acid-neutral conditions on microbial metabolism and therefore on fermentative production has been demonstrated by previous studies as well. Jiang and coll. (2013) found that a pH value between 6 and 7 resulted in an increase of around 20% of the hydrolysis rate, and doubled the VFAs production in the batch reactor compared to the batch reactor with uncontrolled pH [59]. Similarly, Wang and coll. (2014) reported a 17.5 times increase in VFA production, and a 7.5 times increase in VFA yield at a pH value of six compared to a pH 4 [60]. Eryildiz and coll. (2019) and Lukitavesa and coll. (2020) also studied the effect of three different pH (4, 5, 6) on VFA production from citrus waste and reported highest VFA production and yield at pH 6 [21,46].

Figure 2B shows the interaction of C:N ratio and temperature and Figure 2C reflects the interaction of C:N ratio and pH. The RSM indicated that VFA production gradually increased with increasing C:N ratio, whereas the increase in VFA was more abrupt with increasing temperature and pH up to 60 °C and 6, respectively. The C:N ratio is an important parameter in anaerobic fermentation for VFA production and should be taken into consideration. The results obtained by Liu and coll. (2008) showed that the initial C:N ratio was one of the most important factors influencing the distribution patterns of VFAs and the yield of total VFAs [61]. However, there is an optimal narrow range of C:N ratio beyond where there is no further increase in VFA production. This is because it is important for an optimum amount of nitrogen to be present in the feedstock to avoid either nutrient limitation (too low nitrogen) or ammonia toxicity (too high nitrogen) [61,62]. A C:N ratio of 25 was found to be optimum for acetate production in this study. Our results were similar to the results reported by Wang and coll. (2014) who discovered that, when the temperature is increased, an increase was required in the C/N ratio in order to reduce the risk of ammonia inhibition, thus revealing the interactive effect between temperature and C:N ratio [62].

Many optimization studies have been conducted for increasing VFA production from various SOWs [21,46,58]; however, no prior statistical optimization studies (to the best of author’s knowledge) aimed at optimizing acetate concentration in the VFA pool. This is of interest because acetate is the least inhibitory VFA to microbial growth and metabolism and, therefore, can be used in higher concentrations compared to other VFAs. In this study, acetate accounted for 45%–86% of the total VFAs in different experimental runs. The highest fraction of acetate (~86%) was observed in the runs giving the highest acetate concentration in terms of mgL^-1^. Maximum acetate concentration (8061 mg L^−1^) was obtained at pH 6, temperature 60 °C, and a C:N ratio of 25; thus, these conditions can be deemed as optimum conditions according to the developed model. Post-analysis model validation experiment produced 3423 mg L^−1^ and 8057 mg L^−1^ of acetate under optimized and unoptimized conditions, respectively. This marked a 2.4-fold increase in acetate levels. The post-analysis run gave a response value for acetate concentration that was within >95% of the predicted value by the quadratic model indicating the usefulness, and accuracy of the model.

### 3.3. Growth of Yarrowia Lipolytica on Acetate-Rich Anaerobic Fermentation Product Derived from SOW

Considering the industrial and environmental challenges associated with using low-cost waste as starting feedstocks for microbial biochemical production, we employed and developed an acetogenic consortium for a sustainable conversion of SOWs into mainly acetate (and a few other VFAs). The final aim is to use this acetate-rich fermentate as a sole carbon source for growth, metabolism, and biochemical production of itaconic acid by the selected host - *Y.lipolytica*. In this direction, cafeteria waste and corn stover derived fermentate was used to grow *Y.lipolytica* in batch cultures to evaluate the potential of the fermentate for practical application. Anaerobic fermentate was predominantly composed of short-chain (C2-C3) VFAs (~90% of the total substrate) and small amounts of long-chain VFAs (C4-C6) were detected.

**Figure 4:**
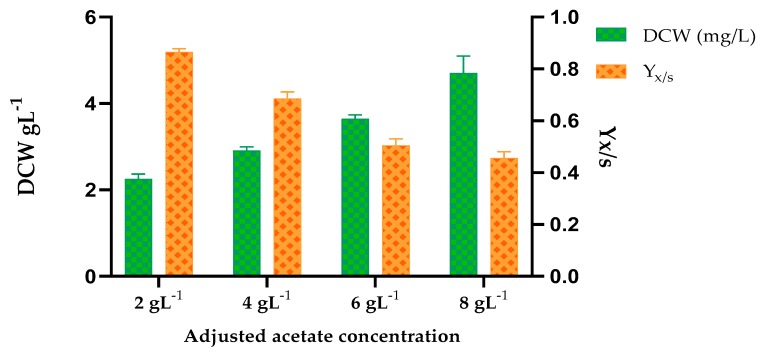
Comparison of dry cell weight (DCW) and growth yield coefficient (Y_X/S_) obtained during batch cultivation on different substrate loading of anaerobic fermentate derived from cafeteria waste and corn stover mixture.

During the experimental observation period of 20 days, batch cultures with four different concentrations of the fermentate (adjusted to achieve acetate concentration of 2, 4, 6, and 8 g/L) showed an increase in biomass density (in term of DCW) with increasing acetate concentration. (Figure 4, Table 6). Since all the concentrations of acetate selected for this study were conducive for cell growth of *Y.lipolytica*, higher initial acetate concentrations could help achieve higher cell densities. Higher acetate concentration can be attained by increasing the organic load of SOW in the anaerobic fermentation process using the thermophilic acetogenic consortium and the optimized parameters of this study. However, an important parameter to be considered for analyzing economical and practical application is the growth yield coefficient, Y_X/S,_ which appeared to decrease when higher concentrations of adjusted acetate levels (6 and 8 g L^−1^) were used (Table 6).

It is established that higher total VFA concentration shows stronger inhibitory effects on growth, and the yeast would require a longer lag phase prior to effective cell growth [63]. According to Rodrigues and Pais (2000), high initial VFA concentrations inhibit cell growth by chemically interfering with the membrane transport of phosphate, thereby increasing ATP expenditure [64]. In this study, even though there was substantial biomass growth at all the concentrations tested, the lag phase differed (Table 6). The general trend was as expected, the lag phase increased with increasing adjusted acetate concentrations of the substrate. Previous studies have shown different upper limits for inhibitory VFA concentrations [60,65,66]. Different systems have their own levels of VFAs that can be considered “normal” for the reactor, and conditions that cause instability in one reactor do not cause problems in another reactor [67]. The inhibitory concentrations also vary with the type of VFA. Literature studies have shown that acetate has the least inhibitory effect on cell growth and metabolism compared to other common VFAs (propionic acid, butyric acid, valeric acid, iso-butyric acid, iso-valeric acid). In a study conducted by Gao and coll. (2017), *Y.lipolytica* showed a preference for acetate and faster utilization rates of acetate over other VFAs when a mixture of VFAs was used as a substrate. Slower utilization rates of other VFAs (propionate and butyrate) compared to acetate can be attributed to their different metabolic fates after intake.

## 4. Conclusions

The present study was conducted to screen the effects of physical and chemical factors and to optimize acetate production by anaerobic fermentation of two SOWs—cafeteria waste and corn stover. The screening of influential factors using two-level factorial design revealed that incubation temperature, BES addition, and shaking conditions (100 rpm) had the most significant effect on acetate production among the 10 factors tested. The ANOVA analysis confirmed that the selected model was significant. The statistical optimization experiment using RSM resulted in a maximum acetate production of ~8000 mg L^−1^ at a temperature, pH, and C:N ratio of 60 °C, 6, and 25, respectively. It is noteworthy that the difference in the three levels selected by the central composite design for the C:N ratio had a considerable difference among them and fine-tuning of this parameter can further improve acetate production as is observed in previous studies. So, further optimization with a narrow range of C:N ratio can be done. Supplementation of methanogen inhibitor, BES, may not be needed if higher substrate loading is used as higher acetate and/or VFA production is known to inhibit methanogenesis. In the absence of acetoclastic methanogenesis, this can lead to further acetate accumulation.

This study also demonstrated the use of acetate-rich fermentate as a sole carbon source (without any nutrient addition) for the growth of an industrially relevant yeast selected for this study—*Y.lipolytica*—which can convert VFAs in the fermentation product (derived from cafeteria waste and corn stover) into higher-value-added products. According to the results of this study, different initial acetate concentrations exerted different inhibitory effects on cell growth which is mainly evident by the different duration of lag phase and biomass production (in terms of DCW). Higher initial acetate concentrations required longer lag phase though the biomass density was not affected significantly. Our future work is being directed toward engineering *Y.lipolytica* to improve acetate uptake and direct its carbon flux toward the heterologous expression of itaconic acid which has widespread industrial applications. Thus, developing an acetogenic consortium and optimizing VFA production for higher acetate concentrations was an important milestone in the direction of using low-cost waste substrate for the production of higher-value biochemicals using *Y.lipolytica* as a host organism. In addition, the acetate-rich fermentate can be used by other industrial microorganisms for the production of various by-products, the feasibility of which can be checked in future studies.

## Figures and Tables

**Table 1:** Factors chosen for the two-level factorial design.

Factor	Name	Units	Type	Minimum ^a^	Maximum ^a^	Mean	Std. Dev.
A	Temperature	°C	Numeric	45.00	60.00	52.50	7.62
B	pH		Numeric	5.00	7.00	6.00	1.02
C	C/N ratio	g VS	Numeric	15.00	25.00	20.00	5.08
D	Shaking	RPM	Categoric	Yes	No	Levels ^b^:	2.00
E	BES		Categoric	Yes	No	Levels ^b^:	2.00
F	NaHCO_3_	g/L	Numeric	2.00	4.00	3.00	1.02
G	NH_4_CL	9/L	Numeric	1.0000	3.00	2.00	1.02
H	MgCl_2_.6H_2_O	g/L	Numeric	0.5000	1.0000	0.7500	0.2540
J	KCL	g/L	Numeric	0.3000	0.6000	0.4500	0.1524
K	Na_2_S.9H_2_O		Categoric	Yes	No	Levels ^b^:	2.00

^a^ Minimum and maximum values presented here were the two levels selected for the experimental design. ^b^ The three categoric variables (Shaking, BES, Na_2_S.9H_2_O) were also tested at two levels depicted as “Yes” and “No” which indicated their presence and absence, respectively.

**Table 2:** Experimental range and levels of independent variables for response surface methodology design.

Codes	Variables			Levels ^a^		
		−1.682	−1	0	1	1.682
A	Temperature	43.18	50	60	70	76.82
B	pH	2.64	4	6	6	9.36
C	C:N ratio	16.59	20	25	30	33.41

^a^ Each variable was tested at 5 discreet values, i.e., levels denoted as +1.682 and −1.682 (axial points), +1 and −1 (factorial points), and 0 (the center point).

**Table 3:** Substrate and inoculum characteristics.

Parameters	Cafeteria Waste	Corn Stover	Inoculum
Total solids (TS, %w/w)	28.3 ± 0.6	94.8 ± 0.6	4.3 ± 0.5
Volatile solids (VS, %w/w) ^b^	23.2 ± 0.7	89.5 ± 0.8	2.6 ± 1
VS /TS (%)	81.9	94.4	60.4
C:N ^b^	15:1	56:1	2.5
Volatile fatty acids/alkalinity	ND	ND	1.3
Lignin (%) ^a^	ND	12.8	ND
Cellulose (%) ^a^	ND	39.4	ND
Hemicellulose (%)^a^	ND	32.1	ND

ND = Not determined; ^a^ Based on total solids of the sample; ^b^ Based on total weight of the sample.

**Table 4:** Central composite design along with actual (experimental) and predicted values of the dependent variable.

Run Order	Temperature (°C)	pH	C:N Ratio	Acetate Conct. (mg L^−^^1^)	
	A	B	C	Actual Value	Predicted Value
1	60	6	25	8011.41	7983.16
2	43.1821	6	25	1939.26	2060.80
3	76.8179	6	25	4726.20	4652.27
4	50	4	30	2929.86	2806.26
5	60	6	25	8015.88	7983.16
6	60	9.36359	25	2050.74	1978.79
7	60	6	25	8061.42	7983.16
8	70	4	30	4468.65	4548.17
9	60	2.63641	25	2361.30	2480.86
10	70	4	20	4706.98	4595.46
11	70	8	20	4010.55	4100.48
12	50	4	20	3015.96	2943.18
13	60	6	33.409	5908.11	5848.53
14	50	8	20	2873.79	2760.60
15	60	6	16.591	5828.55	5935.74
16	60	6	25	7997.34	7983.16
17	60	6	25	7969.41	7983.16
18	50	8	30	2626.32	2704.18
19	70	8	30	4094.58	4133.69
20	60	6	25	7851.69	7983.16

**Table 5:** Analysis of variance (ANOVA) for quadratic model developed for acetate production by the thermophilic acetogenic consortium.

Source	Sum of Squares	df	Mean Square	F-Value	*p*-Value	
**Model ^a^**	1.005 × 10^8^	9	1.116 × 10^7^	753.01	<0.0001	Significant ^b^
A-A	8.107 × 10^6^	1	8.107 × 10^6^	546.85	<0.0001	
B-B	3.043E × 10^5^	1	3.043 × 10^5^	20.53	0.0011	
C-C	9179.67	1	9179.67	0.6192	0.4496	
AB	48795.83	1	48795.83	3.29	0.0997	
AC	4017.07	1	4017.07	0.2710	0.6140	
BC	3239.85	1	3239.85	0.2186	0.6502	
A²	3.856 × 10^7^	1	3.856 × 10^7^	2601.17	<0.0001	
B²	5.963 × 10^7^	1	5.963 × 10^7^	4022.35	<0.0001	
C²	7.876 × 10^6^	1	7.876 × 10^6^	531.32	<0.0001	
**Residual**	1.482 × 10^5^	10	14,824.24			
Lack of Fit	1.226 × 10^5^	5	24,517.16	4.78	0.0556	Not significant
Pure Error	25656.60	5	5131.32			
**Cor Total**	1.006 × 10^8^	19				

^a^ A—temperature; B—pH; C—C:N ratio; df—degree of freedom; Cor—correlation. ^b^ *p* ≤ 0.05: significant and *p* ≥ 0.05: not significant; R^2^ = 0.9985, Adjusted R^2^ = 0.9972, Predicted R^2^ = 0.9899.

**Table 6:** Biomass production of *Y.lipolytica* on solid organic wastes (SOW) (cafeteria waste and corn stover) fermentate.

Adjusted Acetate Concentration	DCW ^a^ (g L^−^^1^)	Yx/s ^b^ (g g^−^^1^)	Lag Phase (h)
2 g L^−^^1^	2.257 ± 109	0.865 ± 0.0127	<3
4 g L^−^^1^	2.864 ± 0.153	0.786 ± 0.025	<3
6 g L^−^^1^	3.647 ± 0.092	0.505 ± 0.036	12
8 g L^−^^1^	4.705 ± 0.394	0.456 ± 0.057	24

^a^ DCW—dry cell weight; ^b^ Yx/s—growth yield coefficient.

## Supplementary Materials

The following are available online at https://www.mdpi.com/2076-2607/8/3/353/s1. Table S1: Parameters, experimental runs and response of two-level fractional factorial design used for screening of influential factors for acetate production by the developed acetogenic consortia, Table S2: Selected Two-level factorial design-Analysis of Variance (ANOVA), Table S3: Selected Two-level factorial design-Fit Statistics, Table S4: RSM Quadratic model–Fit statistics.
