# Supplementary material for: Acetate Production from Cafeteria Wastes and Corn Stover Using a Thermophilic Anaerobic Consortium: A Prelude Study for the Use of Acetate for the Production of Value-Added Products
Source: Microorganisms. 2020 Mar 2;8(3):353. doi: 10.3390/microorganisms8030353 (PMC7143096; doi:10.3390/microorganisms8030353)
Supplement: Supplementary file 1 [file microorganisms-08-00353-s001.pdf]

Table 1. Parameters, experimental runs and response of two-level fractional factorial design used for screening of influential factors for acetate production by the developed acetogenic consortia.

Runs	A	B	C	D	E	F	G	H	I	J	Acetate mgL^{-1}
1	45	5	15	No	No	2	1	1	0.6	No	541
2	60	5	15	No	No	4	3	0.5	0.3	No	1355
3	45	5	25	Yes	No	2	3	0.5	0.6	No	704
4	45	5	15	Yes	Yes	4	3	1	0.6	No	954
5	60	7	15	No	No	2	1	1	0.3	Yes	1376
6	45	5	25	No	No	4	3	1	0.3	Yes	577
7	60	7	15	Yes	Yes	4	3	1	0.3	Yes	1993
8	60	5	15	Yes	No	2	3	1	0.6	Yes	1611
9	60	5	15	Yes	Yes	2	1	0.5	0.3	No	1965
10	45	7	15	Yes	No	2	3	1	0.3	No	682
11	60	7	15	No	Yes	2	3	0.5	0.6	No	1857
12	60	7	25	Yes	No	2	3	0.5	0.3	Yes	1694
13	45	5	15	No	Yes	2	3	0.5	0.3	Yes	845
14	45	7	25	No	Yes	2	3	1	0.6	Yes	836
15	45	7	25	Yes	Yes	4	3	0.5	0.3	No	1067
16	45	7	25	No	No	2	1	0.5	0.3	No	503
17	60	7	15	Yes	No	4	1	0.5	0.6	No	1649
18	45	7	25	Yes	No	4	1	1	0.6	Yes	635
19	45	7	15	No	Yes	4	1	1	0.3	No	875
20	60	5	25	Yes	Yes	4	3	0.5	0.6	Yes	2148
21	60	5	25	Yes	No	4	1	1	0.3	No	1631
22	60	7	25	No	Yes	4	1	0.5	0.3	Yes	1870
23	45	5	25	No	Yes	4	1	0.5	0.6	No	833
24	60	7	25	Yes	Yes	2	1	1	0.6	No	2117
25	45	5	15	Yes	No	4	1	0.5	0.3	Yes	692
26	45	7	15	Yes	Yes	2	1	0.5	0.6	Yes	1021
27	45	5	25	Yes	Yes	2	1	1	0.3	Yes	1163
28	60	5	25	No	No	2	1	0.5	0.6	Yes	1422
29	60	5	15	No	Yes	4	1	1	0.6	Yes	1863
30	60	5	25	No	Yes	2	3	1	0.3	No	1901
31	45	7	15	No	No	4	3	0.5	0.6	Yes	483
32	60	7	25	No	No	4	3	1	0.6	No	1379

Table 2. Selected Two-level factorial design - Analysis of Variance (ANOVA)

Source	Sum of Squares	df	Mean Square	F-value	p-value	
**Model**	9.022E+06	3	3.007E+06	858.80	< 0.0001	significant
A-Temperature	7.431E+06	1	7.431E+06	2121.90	< 0.0001	
D-Shaking	3.220E+05	1	3.220E+05	91.95	< 0.0001	
E-BES	1.270E+06	1	1.270E+06	362.56	< 0.0001	
**Residual**	98051.12	28	3501.83			
**Cor Total**	9.120E+06	31				

The **Model F-value** of 858.80 implies the model is significant. There is only a 0.01% chance that an F-value this large could occur due to noise. **P-values** less than 0.0500 indicates model terms are significant. In this case A (Temperature), D (Shaking), E (BES) are significant model terms. Values greater than 0.1000 indicate the model terms are not significant. If there are many insignificant model terms (not counting those required to support hierarchy), model reduction may improve your model.

Table 3. Selected Two-level factorial design - Fit Statistics

**Std. Dev.**	**59.18**	R^2	**0.9892**
Mean	1257.56	**Adjusted R^2**	0.9881
C.V. %	4.71	**Predicted R^2**	0.9860
		**Adeq Precision**	74.6943

The **Predicted R^2** of 0.9860 is in reasonable agreement with the **Adjusted R^2** of 0.9881; i.e. the difference is less than 0.2. **Adeq Precision** measures the signal to noise ratio. A ratio greater than 4 is desirable. Ratio of 74.694 indicates an adequate signal. This model can be used to navigate the design space.

Table 4. RSM Quadratic model – Fit statistics

**Std. Dev.**	**121.75**	R^2	**0.9985**
Mean	4972.40	**Adjusted R^2**	0.9972
C.V. %	2.45	**Predicted R^2**	0.9899
		**Adeq Precision**	69.7423

Adeq Precision measures the signal to noise ratio. A ratio greater than 4 is desirable. Your ratio of 69.742 indicates an adequate signal. This model can be used to navigate the design space.
